# Preoperative thrombocytosis is a significant unfavorable prognostic factor for patients with resectable non-small cell lung cancer

**DOI:** 10.1186/1477-7819-12-37

**Published:** 2014-02-12

**Authors:** Miso Kim, Hyun Chang, Hee Chul Yang, Yu Jung Kim, Choon-Taek Lee, Jae-Ho Lee, Sanghoon Jheon, Kwhanmien Kim, Jin-Haeng Chung, Jong Seok Lee

**Affiliations:** 1 Department of Internal Medicine, Seoul National University Bundang Hospital, Seoul National University College of Medicine, Seongnam, Korea; 2 Department of Thoracic Surgery, Seoul National University Bundang Hospital, Seoul National University College of Medicine, Seongnam, Korea; 3 Department of Pathology, Seoul National University Bundang Hospital, Seoul National University College of Medicine, Seongnam, Korea; 4 Division of Hematology and Medical Oncology, Department of Internal Medicine, Seoul National University Bundang Hospital, 166 Gumi-ro, Bundang-gu, Seongnam, Gyeonggi-do 463-707, Republic of Korea

**Keywords:** Non-small cell lung cancer, Platelet count, Thrombocytosis, Prognosis, Recurrence, Survival

## Abstract

**Background:**

Previous studies have reported that pretreatment thrombocytosis is associated with poor outcomes in several cancer types. This study was designed to evaluate the prognostic significance of preoperative thrombocytosis in patients with non-small cell lung cancer (NSCLC) who undergo surgery.

**Methods:**

We retrospectively reviewed the records of 199 patients who underwent R0 resection for NSCLC between May 2003 and July 2006 at Seoul National University Bundang Hospital, Seongnam, Korea.

**Results:**

The frequency of preoperative thrombocytosis was 7.5% (15/199). Patients with preoperative thrombocytosis had shorter overall survival (OS, *P* = 0.003) and disease-free survival (DFS, *P* = 0.005) than those without thrombocytosis. In multivariable analysis, patients with preoperative thrombocytosis had a significantly greater risk of death and recurrence than those without preoperative thrombocytosis (risk of death: hazard ratio (HR) 2.98, 95% confidence interval (CI) 1.39 to 6.37, *P* = 0.005; risk of recurrence: HR 2.47, 95% CI 1.22 to 5.01, *P* = 0.012). A tendency towards a shorter OS and DFS was observed in three patients with persistent thrombocytosis during the follow-up period when compared with those of patients who recovered from thrombocytosis after surgery.

**Conclusions:**

Preoperative thrombocytosis was valuable for predicting the prognosis of patients with NSCLC. Special attention should be paid to patients with preoperative and postoperative thrombocytosis.

## Background

Lung cancer is the most common cause of cancer-related death worldwide [1]. Non-small cell lung cancer (NSCLC) accounts for approximately 80% of all lung cancers. There are several known prognostic factors for patients with NSCLC. The most important prognostic factor for NSCLC is tumor stage, with advanced stage disease being associated with the poorest prognosis. Several studies have shown the relationship of pretreatment hematologic abnormalities such as anemia, leukocytosis, or thrombocytosis with adverse outcomes in patients with lung cancer [2-7]. In particular, tumor-related thrombocytosis is frequently observed in patients with advanced stages of various malignancies and many experimental and clinical studies have been performed to explain this phenomenon. However, the mechanism of thrombocytosis in patients with cancer is not yet fully understood. It remains unclear whether thrombocytosis is the final result of advanced malignancies or the direct cause that increases the risk of recurrence and metastasis. This study was designed to evaluate the prognostic value of preoperative thrombocytosis in patients with NSCLC who undergo surgery.

## Methods

### Patients

The medical records of 274 consecutive patients who underwent surgical resection (lobectomy or pneumonectomy) of NSCLC between May 2003 and July 2006 at Seoul National University Bundang Hospital, Seongnam, Korea, were reviewed. Subsequently, 75 patients were excluded from the study because of the following exclusion criteria: 1) synchronous extrapulmonary malignancy; 2) apparent acute inflammatory disease in which the platelet count can rise; and 3) severe liver cirrhosis that may cause the platelet count to decline. The remaining 199 patients who underwent complete resection, either lobectomy or pneumonectomy with mediastinal lymph node sampling, were enrolled in the study. Data on patient demographics, laboratory results, and pathology based on the seventh edition of the American Joint Committee on Cancer staging system were evaluated. The study protocol was reviewed and approved by the Institutional Review Board of the Seoul National University Bundang Hospital. The study was conducted in accordance with the principles of the Declaration of Helsinki.

### Definition

Preoperative white blood cell count, hemoglobin level, and platelet count before invasive diagnostic procedures and within 4 weeks before surgery were determined. Leukocytosis was defined as a white blood cell count of more than 1.0 × 10^4^/mm^3^. A hemoglobin level of less than 13.0 g/dL in men and less than 12.0 g/dL in women was considered as anemia. A platelet count of more than 40 × 10^4^/μL was defined as thrombocytosis, in agreement with other studies.

### Statistical analysis

Overall survival (OS) and disease-free survival (DFS) were defined as the time elapsed between the date of surgery and the date of death and the date of recurrence, respectively. Significant differences in variables in relation to thrombocytosis were tested using chi-square test or Fisher’s exact test, as appropriate. OS and DFS were evaluated using Kaplan–Meier analysis, and comparisons of survival between the two groups were made using the log-rank test.

The relative impact of variables was analyzed by univariate analysis using the Cox proportional hazards regression method. Multivariate analysis was performed using a backward stepwise method. pT and pN stage were excluded from multivariate analysis due to multicollinearity. Variables with clinical significance and statistical significance levels of less than 0.05 were selected for covariate analysis. Variables with a *P* value of more than 0.10 were removed during stepwise analysis. Two-tailed *P* values of less than 0.05 were considered statistically significant. All analyses were performed using IBM SPSS Statistics, version 19.0 (IBM Corporation, Armonk, NY, USA).

## Results

### Patient characteristics

Patient clinicopathological characteristics are summarized in Table 1. The study population was predominantly male (74.9%), with a median age of 65 years (range: 20 to 84 years). Among 199 patients, there were 136 (68.3%) current or past smokers. Adenocarcinoma was the most frequent histologic type (53.8%). Overall, 99 patients had stage I cancer, whereas 100 patients had stage II or III tumors. Seventeen patients received neo-adjuvant chemotherapy. Adjuvant chemotherapy with or without radiotherapy was given to 120 patients after surgical resection. The frequency of preoperative thrombocytosis was 7.5% (15/199).

**Table 1:** The relationship between preoperative thrombocytosis and clinicopathological factors

**Variable**	**Thrombocytosis (-) (%)**	**Thrombocytosis (+) (%)**	***P *** **value**
Age (years)			0.867
≤64	90 (92.8)	7 (7.2)	
>65	94 (92.2)	8 (7.8)	
Gender			0.765
Male	137 (91.9)	12 (8.1)	
Female	47 (94)	3 (6)	
Smoking history			0.003
No	63 (100.0)	0 (0)	
Yes	121 (89.0)	15 (11.0)	
Histology			0.002
ADC	104 (97.2)	3 (2.8)	
SCC	63 (84.0)	12 (16.0)	
Others	17 (100.0)	0 (0)	
Leukocytosis			0.004
(-)	173 (94.0)	11 (6.0)	
(+)	11 (73.3)	4 (26.7)	
Anemia			<0.001
(-)	145 (96.6)	5 (3.4)	
(+)	39 (79.5)	10 (20.5)	
Stage			0.432
I	93 (93.9)	6 (6.1)	
II to III	91 (91.0)	9 (9.0)	
pT			1.000
1	34 (94.4)	2 (5.6)	
2 to 4	150 (92.0)	13 (8.0)	
pN			0.687
0	113 (91.9)	10 (8.1)	
1 to 3	71 (93.4)	5 (6.6)	

ADC, adenocarcinoma; SCC, squamous cell carcinoma.

The relationship between preoperative thrombocytosis and clinicopathological factors is shown in Table 1. Preoperative thrombocytosis did not show a correlation with age, gender, or tumor stage. Smoking history and histological types were associated with preoperative thrombocytosis (*P* = 0.003 and *P* = 0.002, respectively).

### Survival analysis

**Figure 1:**
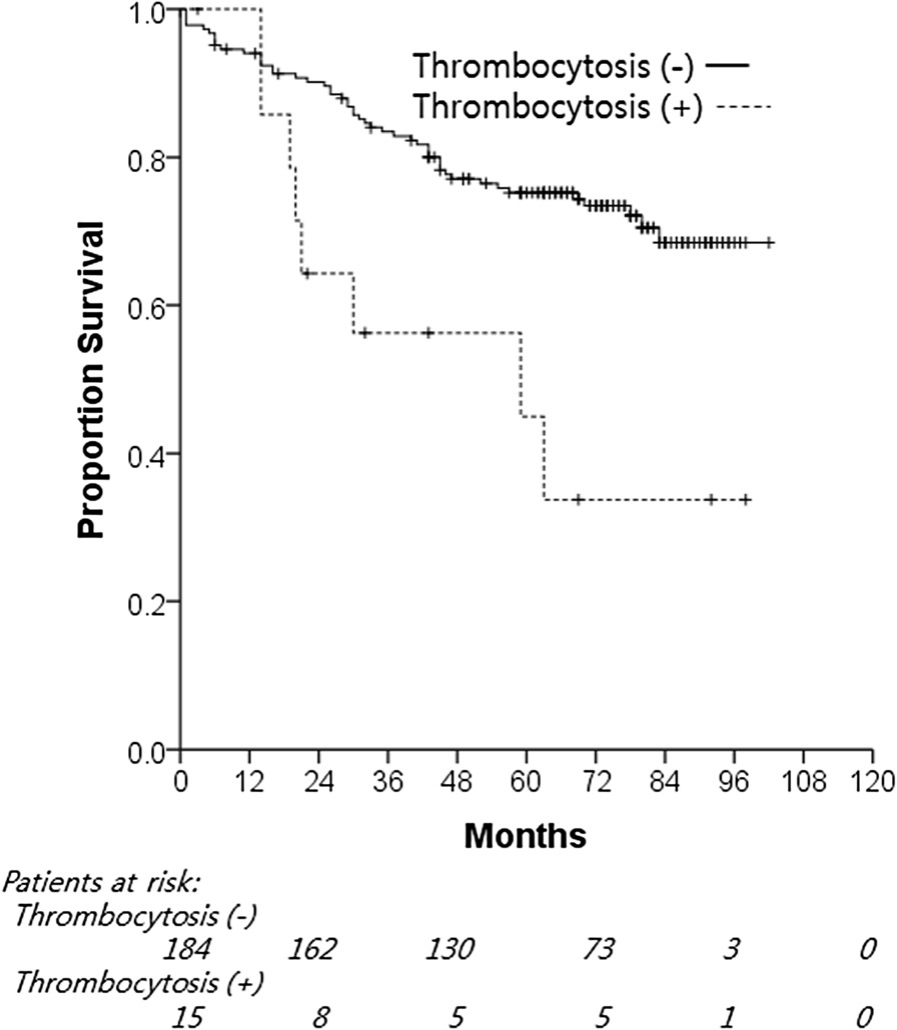
**Correlation between preoperative thrombocytosis and overall survival.** Kaplan–Meier plots of overall survival of patients with resectable NSCLC according to preoperative thrombocytosis (*P* = 0.003). NSCLC, non-small cell lung cancer.

With a median follow-up period of 65 months (range: 0.7 to 102 months), the 5-year OS and DFS rates were 75.2% and 58.3%, respectively. The 5-year OS rate of patients with thrombocytosis was significantly lower than that of patients without thrombocytosis (45.0% versus 75.2%, *P* = 0.003; Figure 1). Univariate analysis identified age (*P* <0.001), smoking history (*P* = 0.030), stage (*P* = 0.037), and preoperative thrombocytosis (*P* = 0.005) as significant prognostic factors for OS. Multivariate analysis confirmed that age (*P* <0.001), stage (*P* = 0.034), and preoperative thrombocytosis (*P* = 0.005) were independent poor prognostic factors for OS (Table 2).

**Table 2:** Univariate and multivariate analysis of prognostic factors for overall survival

**Variable**	**Category**	**Univariate**		**Multivariate**	
		**HR (95% CI)**	***P *** **value**	**HR (95% CI)**	***P *** **value**
Age (years)	>65/≤64	2.99 (1.69 to 5.34)	<0.001	3.03 (1.70 to 5.42)	<0.001
Gender	Female/Male	1.56 (0.81 to 3.00)	0.189	-	-
Smoking	Yes/No	2.03 (1.07 to 3.83)	0.030	-	
Histology	Others/ADC	1.59 (0.94 to 2.68)	0.082	-	-
Stage	II + III/I	1.76 (1.03 to 3.01)	0.037	1.78 (1.04 to 3.05)	0.034
pT	2 to 4/1	2.38 (1.02 to 5.59)	0.046	-	-
pN	1 to 3/0	1.44 (0.86 to 2.43)	0.168	-	-
Leukocytosis	Yes/No	1.34 (0.54 to 3.36)	0.532	-	-
Anemia	Yes/No	1.68 (0.97 to 2.91)	0.066	-	-
Thrombocytosis	Yes/No	2.93 (1.38 to 6.22)	0.005	2.98 (1.39 to 6.37)	0.005

ADC, adenocarcinoma; CI, confidence interval; HR, hazard ratio.

**Figure 2:**
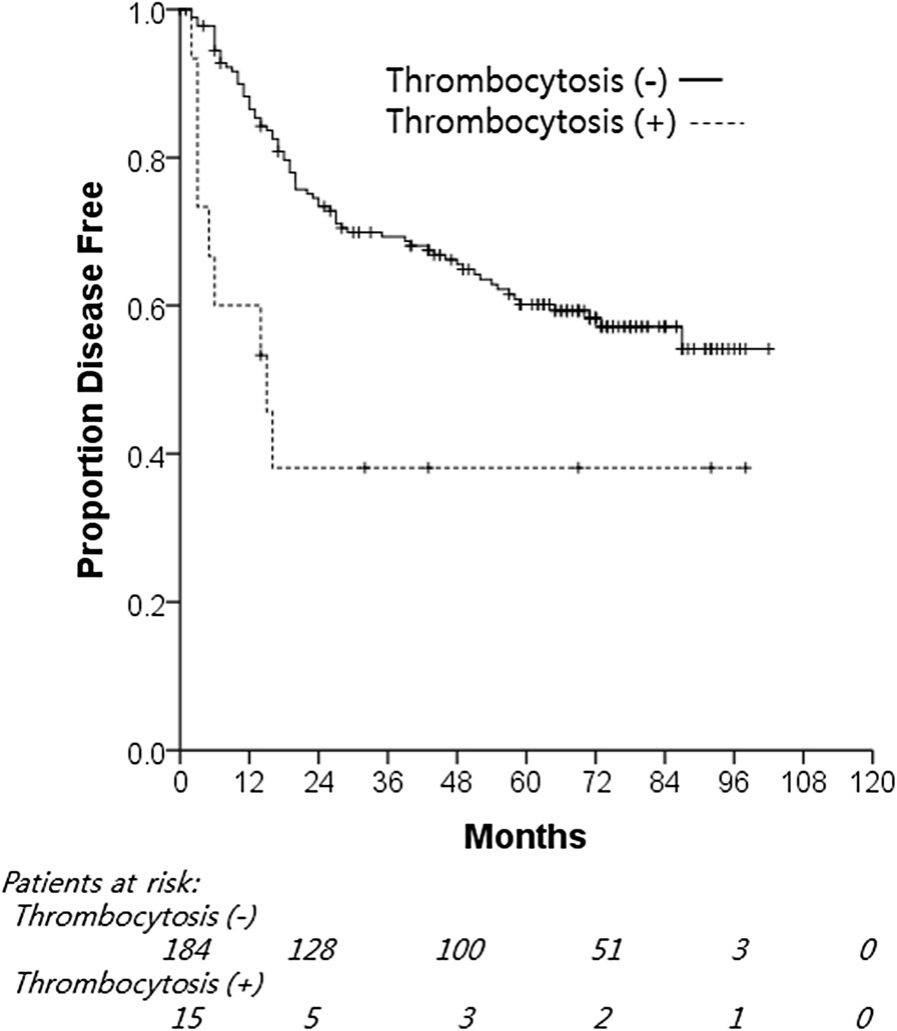
**Correlation between preoperative thrombocytosis and disease-free survival.** Kaplan–Meier plots of disease-free survival of patients with resectable NSCLC according to preoperative thrombocytosis (*P* = 0.005). NSCLC, non-small cell lung cancer.

With a median DFS of 50 months for all patients, the 5-year DFS rate of patients with thrombocytosis was significantly lower than that of patients without thrombocytosis (38.1% versus 68.1%, *P* = 0.005; Figure 2). Eighty-one patients (40.7% of all patients) developed recurrence. Nine (60%) of the 15 patients with thrombocytosis showed recurrence, whereas 73 (40%) of the 184 patients without thrombocytosis showed recurrence. Univariate analysis showed that age, stage, pT, pN, preoperative anemia, and preoperative thrombocytosis were significant prognostic factors for recurrence. Multivariate analyses confirmed that age (*P* = 0.046), stage (*P* <0.001), and preoperative thrombocytosis (*P* = 0.012) were independent prognostic determinants of recurrence (Table 3).

**Table 3:** Univariate and multivariate analysis of prognostic factors for disease-free survival

**Variable**	**Category**	**Univariate**		**Multivariate**	
		**HR (95% CI)**	***P *** **value**	**HR (95% CI)**	***P *** **value**
Age (years)	>65/≤64	1.62 (1.04 to 2.52)	0.034	1.57 (1.01 to 2.46)	0.046
Gender	Female/Male	1.20 (0.72 to 2.00)	0.494	-	-
Smoking	Yes/No	1.14 (0.71 to 1.81)	0.592	-	
Histology	Others/ADC	0.84 (0.76 to 1.85)	0.443	-	-
Stage	II + III/I	2.76 (1.72 to 4.41)	<0.001	2.73 (1.70 to 4.37)	<0.001
pT	2 to 4/1	2.05 (1.05 to 3.98)	0.035	-	-
pN	1 to 3/0	1.82 (1.17 to 2.81)	0.007	-	-
Leukocytosis	Yes/No	1.17 (0.51 to 2.70)	0.706	-	-
Anemia	Yes/No	1.66 (1.04 to 2.64)	0.034	-	-
Thrombocytosis	Yes/No	2.61 (1.33 to 5.24)	0.007	2.47 (1.22-5.01)	0.012

ADC, adenocarcinoma; CI, confidence interval; HR, hazard ratio.

Platelet counts were normalized in 12 out of 15 patients with preoperative thrombocytosis. Among these 12 patients, seven patients recovered from thrombocytosis within 1 month and five patients recovered from thrombocytosis after a few months (median, 1.2 months). Thrombocytosis persisted during the follow-up period in three out of 15 patients. Of these three patients with persistent thrombocytosis, two patients developed systemic recurrence and one had a local relapse after surgery. A tendency towards a shorter OS and DFS was observed in these three patients with persistent thrombocytosis when compared with those of patients who recovered from thrombocytosis (median OS, 63 months versus 15 months, *P* = 0.064; median DFS, 15 months versus 6 months, *P* = 0.142).

## Discussion

Our findings demonstrated that preoperative thrombocytosis in patients with resectable NSCLC was significantly associated with an increased risk of death and disease recurrence. Several previous studies suggested that anemia or leukocytosis in patients with NSCLC might have a negative impact on survival [2-4]. However, our study did not show a relationship between other preoperative hematologic malignancies, namely anemia and leukocytosis, and adverse outcomes. In addition, our study showed that preoperative thrombocytosis was significantly associated with smoking history. Previous studies have shown a positive association between smoking and elevated platelet count [8-10]. Although the exact mechanism by which cigarette smoking induces changes in platelet counts is unclear, the systemic inflammatory response induced by cigarette smoking might contribute to increased platelet counts [11].

A relationship between elevated platelet counts and malignant tumors was initially reported by Reiss *et al*. in 1872 [12]. To date, thrombocytosis has been estimated to occur in approximately 10% to 57% of cancer patients with different solid tumors [13]. In our study, 7.5% of patients with resectable NSCLC had thrombocytosis. The incidence of thrombocytosis in our study was lower than that in previous reports. This may be because we excluded patients with unresectable or metastatic lung cancer from our study.

Correlations between thrombocytosis and shorter survival times have been shown for many solid tumors including gynecological cancers [14-16], gastric cancer [17,18], esophageal cancer [19], rectal cancer [20,21], and lung cancer [3,6,22]. Recently, Kawai *et al*. [21] suggested that the preoperative platelet count before chemoradiotherapy can be a potential predictive marker of response to treatment and of risk of local recurrence after treatment in rectal cancer patients. They hypothesized that platelets may play a pivotal role in the regulation of radio-resistance in colorectal cancer. However, a few studies evaluate the relationship between the prognosis of cancer patients and the post-treatment platelet count. Lee *et al*. [23] demonstrated that thrombocytosis after adjuvant chemotherapy in patients with advanced epithelial ovarian cancer was an independent prognostic factor. In our study, three patients showed persistent thrombocytosis after surgical resection. They had poorer outcomes than did patients in whom platelet levels recovered to normal after surgery.

The mechanism underlying thrombocytosis in cancer patients is not yet fully understood. It is also unclear whether thrombocytosis is a reaction to more aggressive tumors or an active disorder that aggravates cancer progression. Nevertheless, several mechanisms of both tumor-induced platelet activation and platelet-induced cancer progression have been reported. Tumor-related humoral factors, such as granulocyte colony-stimulating factor, interleukin-1, and interleukin-6, may play a role in stimulating megakaryocyte growth and platelet production [24-26]. In addition, recent experimental studies using *in vitro* and *in vivo* murine models have demonstrated that platelets activated by cancer cells may induce the adhesion, growth, and distant metastasis. Platelets provide a procoagulant surface to facilitate cancer-related coagulation; thus, platelet adhesion to the tumor cells may protect the tumor cells from immune responses that will lead to cancer growth and dissemination [27]. Although there is increasing evidence that platelets contribute to tumor progression and metastasis, the molecular mechanisms through which platelets worsen prognosis of cancer patients still need to be studied.

Our study had the limitations of retrospective study, including the relatively small number of patients from a single hospital. Because of the limited number of patients, our results should be interpreted cautiously. In addition, the impact of preoperative and postoperative treatment-related outcome was not evaluated in this study.

## Conclusions

In conclusion, preoperative thrombocytosis is an independent poor prognostic marker for resectable NSCLC. Additionally, our data showed that persistent thrombocytosis may also be a predictive marker for recurrence. Further prospective studies with a larger sample size are required to confirm the prognostic value of preoperative and persistent thrombocytosis in NSCLC patients.

## Abbreviations

ADC: Adenocarcinoma; CI: Confidence interval; DFS: Disease-free survival; HR: Hazard ratio; NSCLC: Non-small cell lung cancer; OS: Overall survival; SCC: Squamous cell carcinoma.

## Competing interests

The authors declare that they have no competing interests.

## Authors’ contributions

MK, HC, and JSL designed this study, collected data, performed analysis, and drafted the manuscript. YJK, CTL, and JHL participated in the study design, literature search, and study coordination. HCY, SJ, and KK collected data and performed data analysis. JHC participated in the study design and helped to draft the manuscript. All authors read and approved the final manuscript.
